# Analysis of differences in the transcriptomic profiles of eutopic and ectopic endometriums in women with ovarian endometriosis

**DOI:** 10.7717/peerj.11045

**Published:** 2021-04-07

**Authors:** Xiao Feng, Lingbin Qi, Xiaoyu Xu, Yun Feng, Xiaoming Gong, Aixingzi Aili, Yu Chen, Zhigang Xue, Jinfeng Xue, Xiaowen Tong

**Affiliations:** 1 Department of Gynecology, Shanghai First Maternity and Infant Hospital, Tongji University School of Medicine, Shanghai, China; 2 Department of Regenerative Medicine, Tongji University School of Medicine, Shanghai, China; 3 Reproductive Medicine Center, Tongji Hospital, Tongji University School of Medicine, Shanghai, China; 4 WOYI OBGYN Medical Group, Beijing, China; 5 School of Life Science and environment, Avans University of Applied Science, Breda, The Netherlands; 6 Department of Obstetrics and Gynecology, Tongji Hospital, Tongji University School of Medicine, Shanghai, China

**Keywords:** Endometriosis, Eutopic endometrial cells, Ectopic endometrial cells, RNAseq, Transcriptomic analysis

## Abstract

**Background:**

Endometriosis is a common gynecological disease among women in their reproductive years. Although much effort has been made, the pathogenesis of this disease and the detailed differences between eutopic endometrial cells and ectopic endometrial cells are still unclear.

**Methods:**

In this study, eutopic and ectopic endometrial cells were collected from patients with and without endometriosis and RNA sequencing was performed. The gene expression patterns and differentially expressed genes (DEGs) in eutopic and ectopic endometrial cells, as well as control endometrial cells, were analyzed using a weighted gene co-expression network analysis (WGCNA) and the DESeq2 package. The functions of significant genes were detected using Gene ontology (GO) enrichment analysis, and qRT-PCR validation was performed.

**Results:**

The results indicated that eight gene modules were found among these three groups. They also indicated that the gene module, which is highly related to eutopic endometrial cells, was mainly enriched in cell adhesion, embryo implantation, etc., while the gene module related to ectopic endometrial cells was mainly enriched in cell migration, etc. The results of differential expression analysis were generally consistent with the WGCNA results through identified significant DEGs between different groups. These DEGs may play an important role in the occurrence of endometriosis, including the infertility associated gene ARNTL and PIWIL2, tissue remodeling gene MMP11, cell survival and migration gene FLT1, inflammatory response gene GNLY, the tumor suppressor genes PLCD1, etc. Further analysis suggested the function of adhesion is stronger in ectopic endometrial cells than in eutopic endometrial cells, while the ectopic endometrium may have a higher potential risk of malignant transformation than eutopic endometrium.

**Conclusions:**

Overall, these data provide a reference for understanding the pathogenesis of endometriosis and its relationship with malignant transformation.

## Introduction

Endometriosis is a common gynecological disease among women in their reproductive years (Zondervan, Becker & Missmer, 2020). It is an estrogen-dependent disorder defined as the growth of endometrial cells outside the uterine cavity, with a chronic inflammatory reaction, associated with pain and infertility (Bulun et al., 2019). The ectopic endometrial tissue is commonly found on the ovaries, fallopian tubes, and peritoneum around the uterus and ovaries. It can also occur in other parts of the body, such as the myometrium, vagina, bladder, rectum, ureter, abdominal wall, nasal cavity, lung, pleura, and brain (Davis & Goldberg, 2017). The ectopic endometrium may implant on the ovarian surface and cause hormone-mediated bleeding and adhesions. Herein, the ovarian endometrioma is formed with an extraovarian pseudocystic structure delineated by the fibrotic tissue (Gordts & Campo, 2019). It may be asymptomatic in its early stages, which makes it difficult to know how common the disease is in the population, but 10% of reproductive-age women are estimated to suffer from endometriosis (Shafrir et al., 2018). However, a reliable diagnosis of endometriosis requires surgical visualization of the lesions (Agarwal et al., 2019) and delays of eight to ten years may occur between onset of symptoms and confirmation of the diagnosis (Ahn, Singh & Tayade, 2017). Endometriosis presents with dysmenorrhea, chronic pelvic pain, dyspareunia, and infertility, which affects the life quality of individual patients and increases the societal burden due to the financial costs and losses in social productivity (Falcone & Flyckt, 2018; Soliman et al., 2017).

Many studies have been conducted on endometriosis, but the etiology and pathogenesis of the disease remain unclear and the pathogenesis of this disease is of great interest for researchers. Most of the research into endometriosis focused on the eutopic endometrium (Poli-Neto et al., 2020). A previous study (Zhao et al., 2017) used RNA sequencing (RNAseq) to analyze differentially expressed genes that were in the eutopic endometrium between women with endometriosis and controls. MMP-11, DUSPI, FOS, SERPINE1, and ADA2 were among the 72 differentially expressed genes (DEGs) related to the pathogenesis of endometriosis, indicating that these genes may be used as biomarkers in the endometrium of women with endometriosis (Zhao et al., 2017). Mortlock et al. (2020) analyzed RNA-seq and genotype data from 206 individuals and indicated that gene expression at 39 loci were associated with endometriosis, including five known endometriosis risk loci. However, the underlying mechanisms causing endometriosis in many of these genetic regions are not yet clear and the difference between the eutopic endometrium and ectopic endometrium and the complex genetic etiology of endometriosis requires further study.

We analyzed endometrial cells from different locations; eutopic cells were collected from the lining of the uterine cavity and ectopic endometrial cells were collected from the ovarian endometriosis cyst walls. RNA-seq was performed, then we analyzed the transcriptomic profiles of cells from the eutopic and ectopic endometria of women with endometriosis and from the eutopic endometrial cells of women without endometriosis. Our study explores the difference between the eutopic endometrium and the ectopic endometrium to better understand the possible mechanisms behind the malignant transformation of the endometriosis.

## Materials and Methods

### Ethical approval

Our study was approved by the institutional ethics committee of Shanghai First Maternity and Infant Hospital (KS16107). All of the subjects included in our study signed an informed consent form before research recruitment.

### Tissue samples

We recruited six women with endometriosis and two women without endometriosis. All subjects had undergone laparoscopic and hysteroscopic surgery during the 8th–11th day of their menstruation, which is the early follicular phase, at Shanghai First Maternity and Infant Hospital in Shanghai, China. Six women with endometriosis were suspected to have ovarian endometriosis and infertility. Among those six women, four of them did not have dysmenorrhea, and the dysmenorrhea visual analogue scale (VAS) of the other two subjects was in the range of 1–2. Two women used as controls were suspected to have tubal infertility with an ovarian follicular cyst more than 4 cm in diameter. Both did not have dysmenorrhea. Ectopic endometrial tissues were obtained by laparoscopy, and eutopic endometrial tissues were achieved by dilation and curettage (D&C). Patients who had any history of acute inflammatory diseases, systemic autoimmune disorders, malignant tumors, or hormone therapies within six months were excluded. Endometriosis was diagnosed by visual confirmation under laparoscopy and pathological analysis. The absence of endometriosis was confirmed by diagnostic laparoscopy.

### Cell samples

All the cryopreserved tissues were minced and incubated with collagenase (Sigma-Aldrich, C2674-100MG) at 37 °C for 10 min, followed by filtration. Endometrial tissues were digested by collagenase/hyaluronidase (Sigma-Aldrich, C2674-100MG/H3506-100MG) at 37 °C for 2 h. After digestion, the endometrial stroma cells and epithelial cells were dispersed. Dozens of cells were mixed and randomly placed into tubes as one group, and 29 groups from eight patients were collected from those tissue samples. Among them, 12 groups were ectopic endometrial cells, 14 groups were eutopic endometrial cells from the patients with ovarian endometriosis, and 3 groups were eutopic endometrial cells from the controls (Table S1).

### Library construction and RNA sequencing

We used the Smart-seq2 protocol with minor modifications due to the low number of cells in each group, which mainly changed the reverse transcriptase for all samples (Picelli et al., 2014). In brief, cells were picked into a lysis buffer to release total RNA and were converted into cDNA using the Oligo-dT30VN primer and Superscript III reverse transcriptase (Invitrogen, 18080044) and further pre-amplified using KAPA HiFi HotStart ReadyMix (Kapa Biosystems, KK2601); AMPure XP beads (Beckman Coulter, A63881) were used to purify the cDNA products and the subsequent tagmentation reaction was performed using Nextera Tagmentation (Illumina, FC-131-1096). The concentration of the cDNA library was adjusted to obtain a total of 5 nM and was sequenced on Illumina HiSeq2500 sequencers in the PE150 model.

### Processing of RNA sequencing data

Raw RNA sequencing reads were trimmed to remove the sequencing adaptor, low-quality reads, and bases using Trimmomatic (V0.33) (Bolger, Lohse & Usadel, 2014). After trimming and quality control, clean reads were aligned to the human genome (GRCh38) using STAR (version 2.7.1a) (Dobin et al., 2013). Uniquely mapped reads were counted with HTSeq and the gene expression level was quantified using the read count.

### Weighted gene co-expression network analysis (WGCNA)

The genes with a mean read count of less than 20 were removed. The read counts of these genes were normalized using the function varianceStablizingTransformation in the DESeq2 package (V1.22.2). Normalized data from each sample in the eutopic endometrial cell group, ectopic endometrial cell group, and control group were used for weighted gene co-expression network analysis (WGCNA) (Langfelder & Horvath, 2008). According to the results from the pickSoftThreshold function, the power was set to 4 to find different gene modules using the blockwiseModules function. Eight gene modules were obtained. The gene co-expression networks of the turquoise and blue modules were extracted using MCODE (version 1.5.1) in Cytoscape (V3.7.2) (Shannon et al., 2003). GO (Gene ontology) enrichment analysis of the hub genes in these two networks was performed using DAVID (version 6.8) (Huang et al., 2007).

### Differential expression analysis

Differential expression analysis among the eutopic endometrial cell group, ectopic endometrial cell group, and control group was performed using the DESeq2 package (V1.22.2). Genes with mean read counts value of less than one were filtered to remove low-expressed features. The differentially expressed genes (DEGs) were calculated using the DESeq function. DEGs were identified with a *p*-value of <0.05 and abs(log2FoldChange) >2. GO (Gene ontology) enrichment analysis of DEGs was performed using DAVID (version 6.8).

### Validation with qRT-PCR

Significantly differentially expressed genes were validated by qRT-PCR using the QuantStudioTM 3 Real-Time PCR System (Applied Biosystems). The primers used in this study are shown in Table S2. All of the genes were normalized to *β*-ACTIN. qRT-PCR was performed in a total reaction volume of 10 µl, including 5 µl 2x TB green premix EX Taq II (Takara, RR820A), 0.4 µl forward primer (10 µmol/L), 0.4 µl reverse primer (10 µmol/L), 3.2 µl DNase-free water, and 1 µl cDNA. qPCR was achieved by heating for two minutes at 95 °C, 40 cycles of 95 °C (5 s), and 60 °C (30 s). We detected for each gene three times, independently.

### Statistical analysis

Our results were analyzed using the ΔΔCT method. An analysis of variance (ANOVA) or t test was conducted using GraphPad Prism 6 to determine the significance among three groups or between two groups, respectively. *p* < 0.05 was considered to be statistically significant.

## Results

### Gene co-expression network analysis of eutopic and ectopic endometrial cells

**Figure 1:**
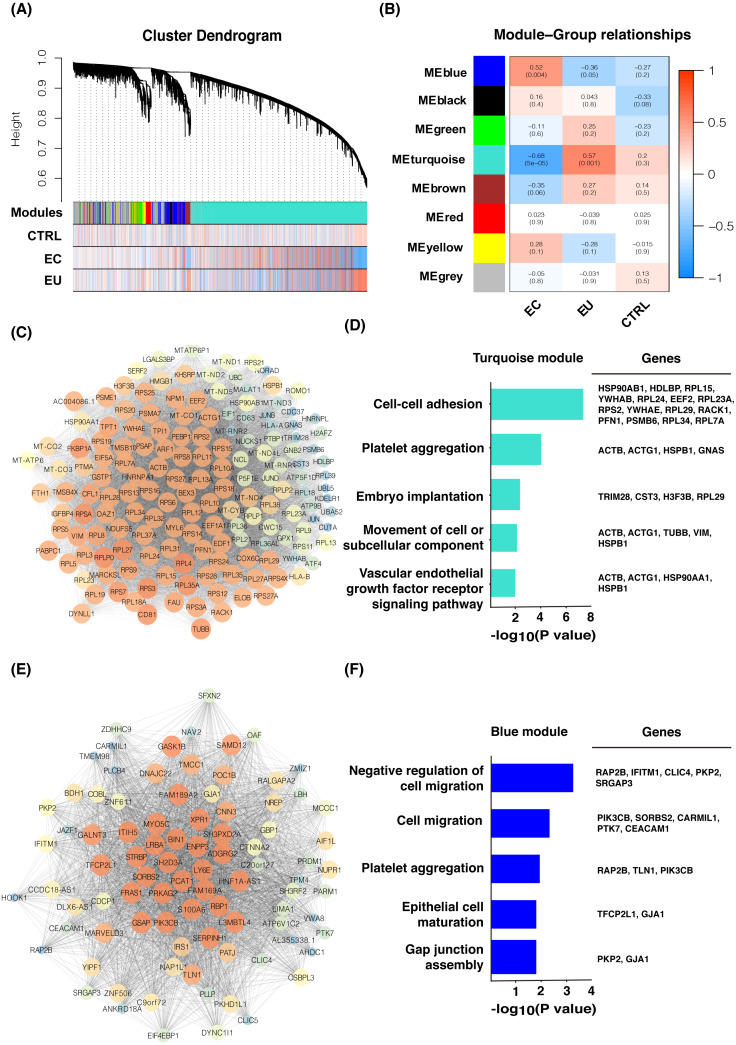
Gene co-expression network and GO analysis of eutopic and ectopic cells. (A) Gene modules were identified by WGCNA and the distribution of gene expression in three groups. (B) Correlation analysis between gene modules and groups. Z summary and *p*-value were presented. (C) Gene co-expression network of hub genes in turquoise module. High MCODE score of genes was mapped to the large size and the bright color of nodes. (D) GO analysis of hub genes of turquoise module, genes enriched in GO terms were presented. (E) Gene co-expression network of hub genes in blue module. High MCODE score of genes was mapped to the large size and the bright color of nodes. (F) GO analysis of hub genes of blue module, genes enriched in GO terms were presented. EU, eutopic endometrial cells group. EC, ectopic endometrial cells group. CTRL, control endometrial cells.

We normalized the read counts of 12 eutopic endometrial cell samples and 14 ectopic endometrial cell samples from women with endometriosis, as well as 3 control endometrial cell samples from healthy women without endometriosis using DESeq2. The genes with a mean read counts value less than 20 were removed and 6,440 genes remained. WGCNA analysis was used and eight gene modules were found related to these three groups (Fig. 1A), including the turquoise module (3,837 genes), blue module (616 genes), brown module (336 genes), yellow module (314 genes), green module (198 genes), red module (150 genes), black module (45 genes), and grey module (944 genes). The eutopic cell group was found to be closely related to the turquoise module according to the correlation analysis (Fig. 1B); the hub genes were enriched in cell adhesion, platelet aggregation, and tumor occurrence by Gene ontology (GO) enrichment analysis. GO terms included cell–cell adhesion, platelet aggregation, embryo implantation, movement of cell or subcellular component, vascular endothelial growth factor receptor signaling pathway, etc. (Figs. 1C and 1D). These genes may play an important role in the pathogenesis of endometriosis. The ectopic endometrial cell group was mainly related to the blue module (Fig. 1B) and the genes were enriched in cell migration, epithelial cell maturation, gap junction assembly, as well as platelet aggregation, etc. (Figs. 1E and 1F), suggesting that genes including PIK3CB, SORBS2, CARMIL1, PTK7, and CEACAM1 may promote cell migration and the formation of eutopic endometrial cells. Genes related to platelet aggregation were found in the blue module, including RAP2B, TLN1, and PIK3CB. These genes cause platelets to aggregate in endometriotic lesions and play critical roles in the development and progression of endometriosis.

### Differential expression analysis among eutopic endometrial cells, ectopic endometrial cells, and control endometrial cells

**Figure 2:**
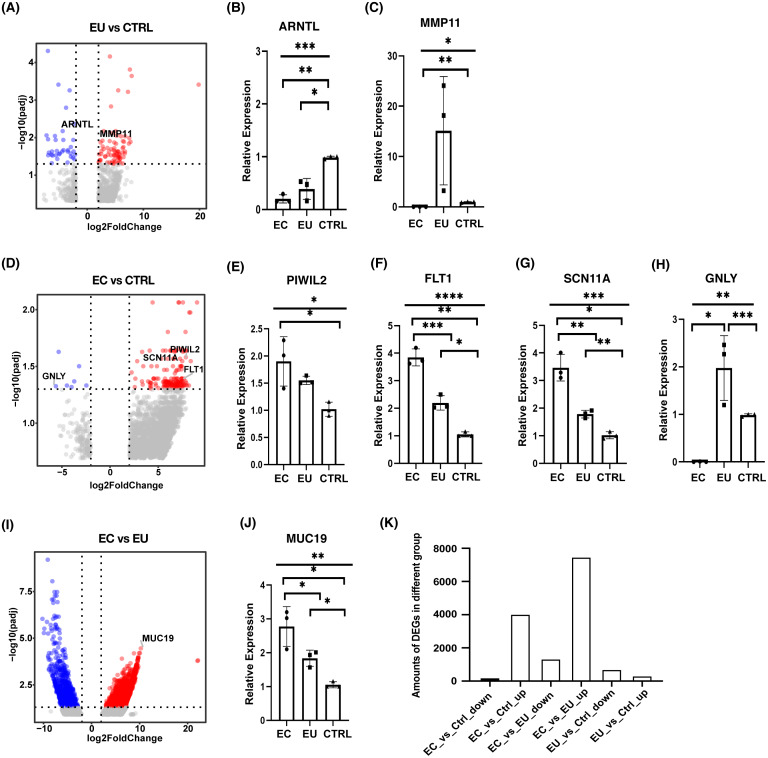
Differential expression analysis among ectopic endometrial cells, eutopic endometrial cells and control endometrial cells. (A) Volcano plot showed DEGs between eutopic endometrial cells and control cells. (B), (C) Bar plots showed the differential expression of ARNTL and MMP11 which were confirmed by qRT-PCR (*n* = 3 independent repeats per group). (D) Volcano plot showed DEGs between ectopic endometrial cells and control cells. (E)–(H) Bar plots showed the differential expression of PIWIL2, FLT1, SCN11A and GNLY which were confirmed by qRT-PCR (*n* = 3 independent repeats per group). (I) Volcano plot showed DEGs between ectopic endometrial cells and eutopic endometrial cells. (J) Bar plot showed the differential expression of MUC19 which was confirmed by qRT-PCR (*n* = 3 independent repeats per group). (K) Bar chart showed the number of overlapping DEGs among three groups.Significant DEGs were identified by abs(Log2foldchange) > 2 and *p*-value > 0.05.

To explore the mechanism of endometriosis, differential expression analysis was performed among the eutopic endometrial cell group, the ectopic endometrial cell group, and the control group using DESeq2. Our results showed that there were 688 genes significantly up-regulated and 298 genes down-regulated in the eutopic endometrial cell group compared with the control group (Fig. 2A). qRT-PCR was used to validate the differentially expressed genes (DEGs) and the results for the down-regulated expression in the eutopic endometrial cell group suggested the defect of ARNTL in the eutopic endometrial cell group may lead to an increased risk of infertility in patients with endometriosis (Fig. 2B, Fig. S1A). MMP11 was significantly up-regulated for the up-regulated expression genes, suggesting that endometrial cells in patients with endometriosis present a mechanical phenotype of tissue remodeling. The increased expression of MMP11 may be the cause of endometrium hyperplasia (Fig. 2C, Fig. S1B).

In addition, there were 155 genes significantly up-regulated and eight genes down-regulated in the ectopic endometrial cell group compared with the control group (Fig. 2D). PIWIL2 was observed, showing an up-regulated expression in ectopic endometrial cells (Fig. 2E, Fig. S1C), suggesting that ectopic endometrial cells were closely related to cell adhesion and deformation. FLT1 plays a very important role in cell migration, chemotaxis, cell survival, angiogenesis regulation, embryonic vasculature development, and tumor cell invasion. FLT1 and SCN11A were up-regulated and GNLY was down-regulated, suggesting that endometriosis is associated with an inflammatory response (Figs. 2F–2H and Figs. S1D–S1F).

Our analysis of differential expression between the ectopic endometrial cell group and the eutopic endometrial cell group showed that there were 7,450 genes up-regulated and 1,327 genes down-regulated in the ectopic endometrial cell group (Fig. 2I). MUC19 was significantly up-regulated in the ectopic endometrial cell group, which indicates that adhesion is stronger in ectopic endometrial cells than in eutopic endometrial cells (Fig. 2J, Fig. S1G). In addition, the numbers of DEGs between ectopic endometrial cells and eutopic endometrial cells were much higher than other groups of DEGs, and many genes were significantly up-regulated in ectopic endometrial cells, regardless of their comparison with eutopic endometrial cells or control cells (Fig. 2K).

### The malignant transformation tendency of endometriosis

**Figure 3:**
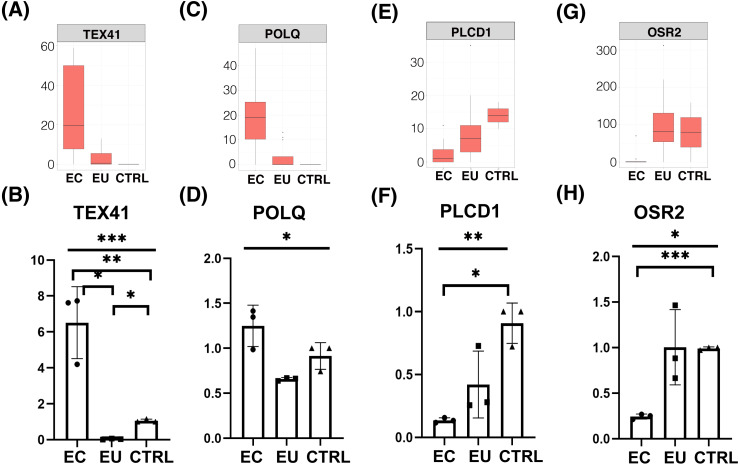
Differential expression of tumor related genes. (A), (C), (E), (G) Box plots showed FPKM expression of tumor genes and tumor suppressor genes in ectopic endometrial cells compare with control cells and eutopic endometrial cells. (B), (D), (F), (H) Box plots showed qRT-PCR of functional DEGs in three groups (*n* = 3 independent repeats per group).

Ovarian endometriosis tissue is known as a benign cyst but the risk of malignant transformation exists. In our analysis, we also saw a considerable number of genes associated with tumorigenesis in endometriosis cells. The results of WGCNA showed that the turquoise and blue modules, which are highly related to the eutopic endometrial cell group and the ectopic endometrial cell group, all contained tumor related genes, such as UBC, UBA52, RPS27A, PIK3CB, IRS1, and CEACAM1 (Figs. 1C and 1E). Whereas, in the differentially expressed gene analysis, the tumor related genes TEX41, POLQ, and FLT1 were highly expressed in the ectopic endometrial cell group of endometriosis, and the expression of tumor suppressor gene PLCD1 was down-regulated when compared with the control group (Figs. 3A–3F, 2F and S1D). These results suggested that the endometrium has tumor-like characteristics, such as implantation, metastasis, and recurrence. Notably, in the ectopic endometrial cell group of endometriosis, tumor suppressor genes such as OSR2 were down-regulated compared with the eutopic endometrial cell group (Figs. 3G and 3H).

## Discussion

We compared the transcriptomic profiles among eutopic endometrial cells, ectopic endometrial cells, and control endometrial cells. WGCNA analysis revealed that hub genes in the eutopic endometrial cell group were mainly enriched in cell–cell adhesion, inflammatory immune response, embryo implantation, etc., and hub genes in the ectopic endometrial cell group were mainly enriched in cell migration, platelet aggregation, tumorigenesis, etc. The results may explain the characteristics of the two different groups of endometriosis. Further analysis of differentially expressed genes confirmed the results of WGCNA. Endometriotic cells were also found especially in the ectopic endometrial cells, and the expression of tumor associated genes was up-regulated and tumor suppression genes were down-regulated.

Infertility is one of the three main symptoms of endometriosis, therefore the genes which are related to infertility are of great importance. ARNTL is one of the putative clock-controlled genes, which supports oocyte fertilization, early embryo development, and implantation potential (Xu et al., 2016). A ARNTL defect in an eutopic endometrial cell group may lead to an increased risk of infertility in patients with endometriosis. In our DEGs analysis, ARNTL was detected as being significantly down-regulated in both the eutopic and ectopic endometrial cell groups compared with the control group. ARNTL is one of the circadian genes, which are highly expressed in the ovaries where they regulate ovulation. A study showed that reduced expression of ARNTL may contribute to a circadian disruption which may be associated with the risk of endometriosis (Jim et al., 2015) and infertility. Prior studies suggested that the role of MMP11 is unique, and includes tissue remodeling, progesterone sensitivity, and the promotion of tumor development (Itoh et al., 2012), and is correlated with poor outcomes in different estrogen-dependent cancers (Nasu et al., 2001; Callegari, Ferguson-Gottschall & Gibori, 2005). Our study in endometrial cells from women with endometriosis further supports those results and suggests ARNTL and MMP11 may play an important role in the pathogenesis of endometriosis.

The mechanism behind the genesis of endometriosis is complicated. John Sampson’s theory of retrograde menstruation is most widely accepted, which postulates that shed endometrial cells or tissues are refluxed through the fallopian tubes to the pelvis or thoracic cavity during menstruation and lead to the development of endometriosis (Sampson, 1927). Mortlock et al. studied the eutopic endometrial tissue from women with and without endometriosis and identified that potential targeted genes were associated with reproductive traits (Mortlock et al., 2020). These results were confirmed in our study. Poli-Neto et al. also studied eutopic endometrial tissue from women with different stages of endometriosis compared to a control. They found the cellular microenvironments and immune cells profiles were different among these tissues (Poli-Neto et al., 2020). For women with endometriosis, the ectopic endometrial tissue may attach to and invade the tissues or organs in the pelvis, such as ovaries and the peritoneal mesothelium. Cell–cell and cell–matrix interactions lead to the attachment, migration, and invasion of the basement membrane, which are the key steps in the complex process that causes endometriosis. In this study, we reported the gene expression pattern among eutopic, ectopic, and control endometrial cells. Our results also confirmed that the enhancement of cell adhesion is the common characteristic in eutopic and ectopic endometrium. Furthermore, compared with eutopic endometrial cells, ectopic endometrial cells were more able to migration.

Increasing amounts of data show that the endometrial lesion is essentially repeated tissue injury and repair of the wound (Guo et al., 2015; Ding et al., 2015; Guo, 2018). Therefore, platelet aggregation in ectopic tissues of women with endometriosis plays a key role in the development and progression of endometriosis. Activated platelets upregulate vascular endothelial growth factor (VEGF) and matrix metalloproteinase 9 (MMP9) to induce angiogenesis (Zhang et al., 2016). Some data show that women with endometriosis are hypercoagulable (Wu et al., 2015; Ding, Liu & Guo, 2018). In our WGCNA results, there was a set of agglutination-related genes, including RAP2B, TLN1, and PIK3CB, in the blue module related to the ectopic endometrial cell group, and DEGs analysis of the ectopic endometrial cell group to the eutopic endometrial cell group showed that the AC068631.1 gene was significantly up-regulated in the ectopic endometrial cell group. This suggests the ectopic endometrial cell group had stronger platelet aggregation characteristics when compared to the eutopic endometrial cell group. Platelet aggregation may play a critical role in the development and progression of endometriosis.

Although endometriosis is a common benign gynecologic disease, accumulated data support evidence of a correlation between endometriosis and ovarian cancer, specifically the two histologic subtypes: endometrioid cancer and ovarian clear cell cancer (Nishida et al., 2000; Modesitt et al., 2002; Oral et al., 2003; Melin et al., 2006; Munksgaard & Blaakaer, 2012; Acien et al., 2015). One study supported the concept that endometriosis is a malignant transformation and that the histogenesis of endometriosis is dependent on several factors, including genetic alterations, hormonal, and immunological factors (Pavone & Lyttle, 2015). We found that the tumor genes, including TEX41, POLQ, and FLT1 were expressed through up-regulation, and tumor suppression genes such as PLCD1 and OSR2 were expressed through down-regulation in the ectopic endometrial cell group, suggesting ectopic endometrial cells may have more risk of malignant transformation than eutopic endometrial cells. Surgical treatment is recommended for endometriosis patients, especially women with ovarian endometriosis when necessary, to reduce the risk of malignant transformation (Torre et al., 2018). Future research should focus on identifying the patient population that would benefit from earlier endometriosis treatment to prevent malignant transformation.

## Conclusions

We analyzed the transcriptome of endometriosis, explored the potential molecular mechanisms of the eutopic and ectopic endometrial cells in cell–cell adhesion, proliferation, migration, etc., and revealed that endometriotic cells, especially ectopic endometrial cells, have a higher tendency of malignant transformation. Our data contributes to the understanding of the pathogenesis of endometriosis and its relationship with tumors.

##  Supplemental Information

10.7717/peerj.11045/supp-1 Table S1 Classification of endometrial cells

10.7717/peerj.11045/supp-2 Table S2 Information of the Primers for qRT-PCR

10.7717/peerj.11045/supp-3 Figure S1 Relative expression of functional DEGs among ectopic endometrial cells, eutopic endometrial cells and control endometrial cells using FPKM valule (A), (B) Box plots showed the differential expression of ARNTL and MMP11 which were calculated by FPKM value. (C)-(F) Box plots showed the differential expression of PIWIL2, FLT1, SCN11A and GNLY which were calculated by FPKM value. (G) Box plot showed the differential expression of MUC19 which was calculated by FPKM value. Significant DEGs were identified by abs(Log2foldchange) > 2 and *p*-value > 0.05.

10.7717/peerj.11045/supp-4 Supplemental Information 4 Flowchart of the Research

## Additional Information and Declarations

### Data Availability

The following information was supplied regarding data availability: Raw data are available at Genome Sequence Archive for Human (GSA-Human), accession number: HRA000663.

## References

[ref-1] Acien P, Velasco I, Acien M, Capello C, Vela P (2015). Epithelial ovarian cancers and endometriosis. Gynecologic and Obstetric Investigation.

[ref-2] Agarwal SK, Chapron C, Giudice LC, Laufer MR, Leyland N, Missmer SA, Singh SS, Taylor HS (2019). Clinical diagnosis of endometriosis: a call to action. American Journal of Obstetrics and Gynecology.

[ref-3] Ahn SH, Singh V, Tayade C (2017). Biomarkers in endometriosis: challenges and opportunities. Fertility and Sterility.

[ref-4] Bolger AM, Lohse M, Usadel B (2014). Trimmomatic: a flexible trimmer for Illumina sequence data. Bioinformatics.

[ref-5] Bulun SE, Yilmaz BD, Sison C, Miyazaki K, Bernardi L, Liu S, Kohlmeier A, Yin P, Milad M, Wei JJ (2019). Endometriosis. Endocrine Reviews.

[ref-6] Callegari EA, Ferguson-Gottschall S, Gibori G (2005). PGF2alpha induced differential expression of genes involved in turnover of extracellular matrix in rat decidual cells. Journal of Reproductive Biology and Endocrinology.

[ref-7] Davis AC, Goldberg JM (2017). Extrapelvic endometriosis. Seminars in Reproductive Medicine.

[ref-8] Ding D, Liu X, Duan J, Guo SW (2015). Platelets are an unindicted culprit in the development of endometriosis: clinical and experimental evidence. Human Reproduction.

[ref-9] Ding D, Liu X, Guo SW (2018). Further evidence for hypercoagulability in women with ovarian endometriomas. Reproductive Sciences.

[ref-10] Dobin A, Davis CA, Schlesinger F, Drenkow J, Zaleski C, Jha S, Batut P, Chaisson M, Gingeras TR (2013). STAR: ultrafast universal RNA-seq aligner. Bioinformatics.

[ref-11] Falcone T, Flyckt R (2018). Clinical management of endometriosis. Obstetrics and Gynecology.

[ref-12] Gordts S, Campo R (2019). Modern approaches to surgical management of endometrioma. Best Practice & Research: Clinical Obstetrics & Gynaecology.

[ref-13] Guo SW (2018). Fibrogenesis resulting from cyclic bleeding: the Holy Grail of the natural history of ectopic endometrium. Human Reproduction.

[ref-14] Guo SW, Ding D, Shen M, Liu X (2015). Dating endometriotic ovarian cysts based on the content of cyst fluid and its potential clinical implications. Reproductive Sciences.

[ref-15] Huang DW, Sherman BT, Tan Q, Kir J, Liu D, Bryant D, Guo Y, Stephens R, Baseler MW, Lane HC, Lempicki RA (2007). DAVID Bioinformatics Resources: expanded annotation database and novel algorithms to better extract biology from large gene lists. Nucleic Acids Research.

[ref-16] Itoh H, Kishore AH, Lindqvist A, Rogers DE, Word RA (2012). Transforming growth factor *β*1 (TGF *β*1) and progesterone regulate matrix metalloproteinases (MMP) in human endometrial stromal cells. Journal of Clinical Endocrinology and Metabolism.

[ref-17] Jim HS, Jim HS, Lin H, Tyrer J, Lawrenson K, Dennis J, Chornokur G, Chen Z, Chen AY, Permuth-Wey J, Aben KK, Anton-Culver H, Antonenkova N, Bruinsma F, Bandera E, Bean Y, Beckmann M, Bisogna M, Bjorge L, Bogdanova N, Brinton L, Brooks-Wilson A, Bunker C, Butzow R, Campbell I, Carty K, Chang-Claude J, Cook L, Cramer D, Cunningham J, Cybulski C, Dansonka-Mieszkowska A, Du Bois A, Despierre E, Sieh W, Doherty J, Dörk T, Dürst M, Easton D, Eccles D, Edwards R, Ekici A, Fasching P, Fridley B, Gao Y, Gentry-Maharaj A, Giles G, Glasspool R, Goodman M, Gronwald J, Harter P, Hasmad H, Hein A, Heitz F, Hildebrandt M, Hillemanns P, Hogdall C, Hogdall E, Hosono S, Iversen E, Jakubowska A, Jensen A, Ji B, Karlan B, Kellar M, Kiemeney L, Krakstad C, Kjaer S, Kupryjanczyk J, Vierkant R, Lambrechts D, Lambrechts S, Le N, Lee A, Lele S, Leminen A, Lester J, Levine D, Liang D, Lim BK, Lissowska J, Lu K, Lubinski J, Lundvall L, Massuger L, Matsuo K, McGuire V, McLaughlin J, McNeish I, Menon U, Milne R, Modugno F, Thomsen L, Moysich K, Ness R, Nevanlinna H, Eilber U, Odunsi K, Olson S, Orlow I, Orsulic S, Palmieri Weber R, Paul J, Pearce CL, Pejovic T, Pelttari LM, Pike MC, Poole EM, Schernhammer E, Risch HA, Rosen B, Anne Rossing M, Rothstein JH, Rudolph A, Runnebaum IB, Rzepecka IK, Salvesen HB, Schwaab I, Shu X-O, Shvetsov YB, Siddiqui N, Song H, Southey MC, Spiewankiewicz B, Sucheston-Campbell L, Teo S-H, Terry KL, Thompson PJ, Tangen IL, Tworoger SS, Van Altena AM, Vergote I, Walsh CS, Wang-Gohrke S, Wentzensen N, Whittemore AS, Wicklund KG, Wilkens LR, Wu AH, Wu X, Woo Y-L, Yang H, Zheng W, Ziogas A, Amankwah E, Berchuck A, Schildkraut JM, Kelemen LE, Ramus SJ, Monteiro ANA, Goode EL, Narod SA, Gayther SA, Pharoah PDP, Sellers TA, Phelan CM, Georgia Chenevix-Trench on behalf of the AOCS management group (2015). Common genetic variation in circadian rhythm genes and risk of epithelial Ovarian Cancer (EOC). Journal of Genetics and Genome Research publishes.

[ref-18] Langfelder P, Horvath S (2008). WGCNA: an R package for weighted correlation network analysis. BMC Bioinformatics.

[ref-19] Melin A, Sparen P, Persson I, Bergqvist A (2006). Endometriosis and the risk of cancer with special emphasis on ovarian cancer. Human Reproduction.

[ref-20] Modesitt SC, Tortolero-Luna G, Robinson JB, Gershenson DM, Wolf JK (2002). Ovarian and extraovarian endometriosis-associated cancer. Obstetrics and Gynecology.

[ref-21] Mortlock S, Kendarsari RI, Fung JN, Gibson G, Yang F, Restuadi R, Girling JE, Holdsworth-Carson SJ, Teh WT, Lukowski SW, Healey M, Qi T, Rogers PAW, Yang J, McKinnon B, Montgomery GW (2020). Tissue specific regulation of transcription in endometrium and association with disease. Human Reproduction.

[ref-22] Munksgaard PS, Blaakaer J (2012). The association between endometriosis and ovarian cancer: a review of histological, genetic and molecular alterations. Gynecologic Oncology.

[ref-23] Nasu K, Kai K, Fujisawa K, Takai N, Nishida Y, Miyakawa I (2001). Expression of cathepsin L in normal endometrium and endometrial cancer. European Journal of Obstetrics & Gynecology and Reproductive Biology.

[ref-24] Nishida M, Watanabe K, Sato N, Ichikawa Y (2000). Malignant transformation of ovarian endometriosis. Gynecologic and Obstetric Investigation.

[ref-25] Oral E, Ilvan S, Tustas E, Korbeyli B, Bese T, Demirkiran F, Arvas M, Kosebay D (2003). Prevalence of endometriosis in malignant epithelial ovary tumours. European Journal of Obstetrics & Gynecology and Reproductive Biology.

[ref-26] Pavone ME, Lyttle BM (2015). Endometriosis and ovarian cancer: links, risks, and challenges faced. International Journal of Women’s Health.

[ref-27] Picelli S, Faridani OR, Björklund AK, Winberg G, Sagasser S, Sandberg R (2014). Full-length RNA-seq from single cells using Smart-seq2. Nature Protocols.

[ref-28] Poli-Neto OB, Meola J, Rosa-E-Silva JC, Tiezzi D (2020). Transcriptome meta-analysis reveals differences of immune profile between eutopic endometrium from stage I-II and III-IV endometriosis independently of hormonal milieu. Scientific Reports.

[ref-29] Sampson JA (1927). Metastatic or embolic endometriosis, due to the menstrual dissemination of endometrial tissue into the venous circulation. American Journal of Pathology.

[ref-30] Shafrir AL, Farland LV, Shah DK, Harris HR, Kvaskoff M, Zondervan K, Missmer SA (2018). Risk for and consequences of endometriosis: a critical epidemiologic review. Best Practice & Research: Clinical Obstetrics & Gynaecology.

[ref-31] Shannon P, Markiel A, Ozier O, Baliga NS, Wang JT, Ramage D, Amin N, Schwikowski B, Ideker T (2003). Cytoscape: a software environment for integrated models of biomolecular interaction networks. Genome Research.

[ref-32] Soliman AM, Coyne KS, Gries KS, Castelli-Haley J, Snabes MC, Surrey ES (2017). The effect of endometriosis symptoms on absenteeism and presenteeism in the workplace and at home. Journal of Managed Care & Specialty Pharmacy.

[ref-33] Torre LA, Trabert B, DeSantis CE, Miller KD, Samimi G, Runowicz CD, Gaudet MM, Jemal A, Siegel RL (2018). Ovarian cancer statistics. CA: A Cancer Journal for Clinicians.

[ref-34] Wu Q, Ding D, Liu X, Guo SW (2015). Evidence for a hypercoagulable state in women with ovarian endometriomas. Reproductive Sciences.

[ref-35] Xu J, Li Y, Wang Y, Xu Y, Zhou C (2016). Loss of Bmal1 decreases oocyte fertilization, early embryo development and implantation potential in female mice. Zygote.

[ref-36] Zhang Q, Duan J, Liu X, Guo SW (2016). Platelets drive smooth muscle metaplasia and fibrogenesis in endometriosis through epithelial-mesenchymal transition and fibroblast-to-myofibroblast transdifferentiation. Molecular and Cellular Endocrinology.

[ref-37] Zhao L, Gu C, Ye M, Zhang Z, Han W, Fan W, Meng Y (2017). Identification of global transcriptome abnormalities and potential biomarkers in eutopic endometria of women with endometriosis: a preliminary study. Biomedical Reports.

[ref-38] Zondervan KT, Becker CM, Missmer SA (2020). Endometriosis. New England Journal of Medicine.

