# Supplementary material for: Analysis of differences in the transcriptomic profiles of eutopic and ectopic endometriums in women with ovarian endometriosis
Source: PeerJ. 2021 Apr 7;9:e11045. doi: 10.7717/peerj.11045 (PMC8035894; doi:10.7717/peerj.11045)
Supplement: Table S1 [file peerj-09-11045-s001.docx]

**Supplementary Table 1 Classification of endometrial cells**

|  | eutopic endometrial cell group (n) | ectopic endometrial cell group (n) | Total  (n) |
| --- | --- | --- | --- |
| ovarian endometriosis | 14 | 12 | 26 |
| control | 3 | 0 | 3 |
| Total | 17 | 12 | 29 |
