# Supplementary material for: Analysis of differences in the transcriptomic profiles of eutopic and ectopic endometriums in women with ovarian endometriosis
Source: PeerJ. 2021 Apr 7;9:e11045. doi: 10.7717/peerj.11045 (PMC8035894; doi:10.7717/peerj.11045)
Supplement: Table S2 [file peerj-09-11045-s002.docx]

**Supplementary Table 2 Primers**

| **Genes** | **Primer Name** | **Primer sequence (5’- 3’)** |
| --- | --- | --- |
| β-actin | Forward Primer | CACCCAGCACAATGAAGATCAAGAT |
|  | Reverse Primer | CCAGTTTTTAAATCCTGAGTCAAGC |
| ARNTL | Forward Primer | TACTGTGCTAAGGATGGCTGTTCA |
|  | Reverse Primer | CTGCCCTGAGAATGAGGTGTT |
| MMP11 | Forward Primer | GAGAAGACGGACCTCACCTACAG |
|  | Reverse Primer | ATCCCCTTCTCGGTGAGTCTT |
| GNLY | Forward Primer | GAAGGGAGAGTGGATTTGGCT |
|  | Reverse Primer | TGGGCTTATCCACCATCTTCTTC |
| PIWIL2 | Forward Primer | GGTGGATTTCTATCTTCTTGCCCATCA |
|  | Reverse Primer | GTACATGTGGCACAGTTTGAAAGTCAG |
| FLT1 | Forward Primer | CACCCCTGTAACCATAATCATTCCGAA |
|  | Reverse Primer | CTTATGTTTCTTCCCACAGTCCCAACT |
| SCN11A | Forward Primer | CCATGAAATCCATCCTTGACCATCTCA |
|  | Reverse Primer | TTGGTGAAGTAGTATTGCCTCAAAGCA |
| ADGB | Forward Primer | ATTGACCAAGAAGAGCGGTTGAAGTTA |
|  | Reverse Primer | TTTGTTTCTGTACTCTTCCCGGATGTC |
| MARCKSL1 | Forward Primer | TGGCCACGTGAAAAGCAATG |
|  | Reverse Primer | CAGAAGAATCACCCCCACCC |
| MUC19 | Forward Primer | GGTGATTCAAGTGCAAGAAATGGGTTT |
|  | Reverse Primer | TCCAGAATCAGAAGACCAAGTGTTTCC |
| TEX41 | Forward Primer | AGTTTCCCTGCCCACATCTTT |
|  | Reverse Primer | TGAGGCAGAGTGAGTCCAGAAG |
| POLQ | Forward Primer | AGGAATGGTGGTTGTGGATGA |
|  | Reverse Primer | GGCGAAAGTCGGTATGGTAGA |
| VSX2 | Forward Primer | TTCAACGAAGCCCACTACCC |
|  | Reverse Primer | CCTCCAGCGACTTTTTGTGC |
| ZNF487 | Forward Primer | CTTTGTAAGGAACCCTGCTGAGTGTAA |
|  | Reverse Primer | AGACGGCTTTCCCATTTCCATCATATT |
| PLCD1 | Forward Primer | GTGACCACTCCCAGACAGACT |
|  | Reverse Primer | AAGCCGTCCTTGGTCATCTG |
| EGR1 | Forward Primer | ATCCTTTCCTCACTCGCCCA |
|  | Reverse Primer | CTCGTTGTTCAGAGAGATGTCAGG |
| OSR2 | Forward Primer | GGCAGACACTTTACCAAATCCTAC |
|  | Reverse Primer | CACTCCTGACATTTGAAGGGTTTT |
