# Supplementary figures and images for: Analysis of differences in the transcriptomic profiles of eutopic and ectopic endometriums in women with ovarian endometriosis
Source: PeerJ. 2021 Apr 7;9:e11045. doi: 10.7717/peerj.11045 (PMC8035894; doi:10.7717/peerj.11045)

**(A)**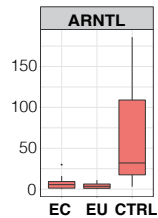**(B)**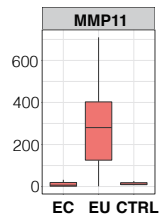**(C)**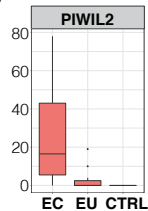**(D)**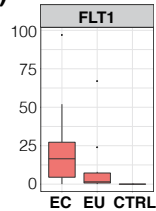**(E)**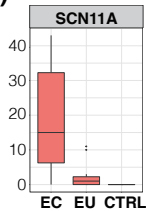**(F)**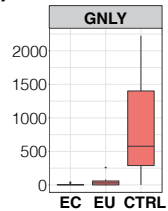**(G)**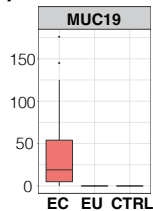

Supplement: Figure S1 — (A), (B) Box plots showed the differential expression of ARNTL and MMP11 which were calculated by FPKM value. (C)-(F) Box plots showed the differential expression of PIWIL2, FLT1, SCN11A and GNLY which were calculated by FPKM value. (G) Box plot showed the differential expression of MUC19 which was calculated by FPKM value. Significant DEGs were identified by abs(Log2foldchange) > 2 and p-value > 0.05. [file peerj-09-11045-s003.pdf]

**
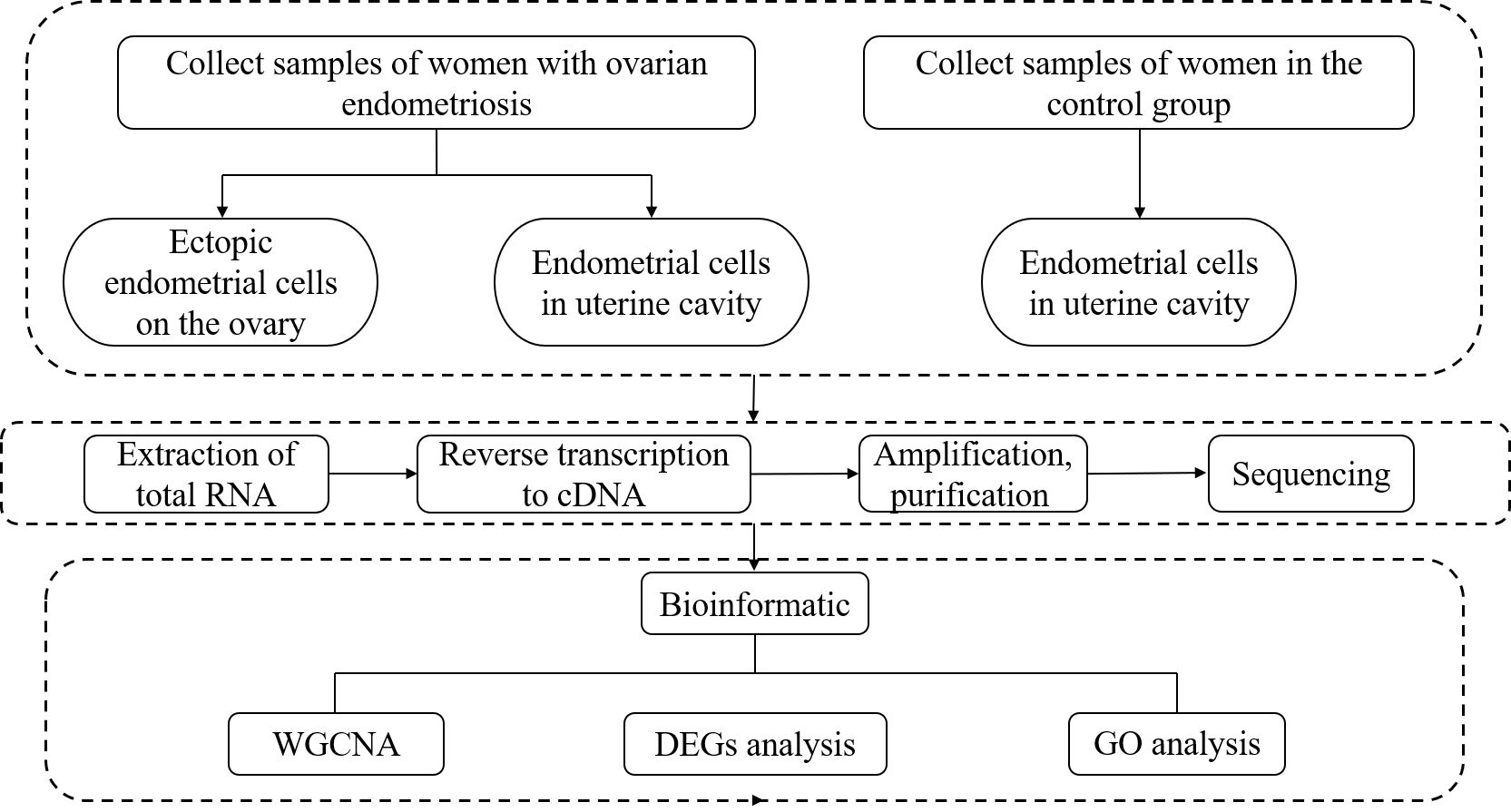
**

Supplementary flowchart

Supplement: Supplemental Information 4 [file peerj-09-11045-s004.docx]
